# Development and Prospective Pilot Validation of the Stanley Bile Duct Injury-Repair Risk Stratification Score for Definitive Biliary Reconstruction After Post-cholecystectomy Bile Duct Injury

**DOI:** 10.7759/cureus.114984

**Published:** 2026-08-22

**Authors:** Purushothaman Suresh, Saravanan J, Satish Devakumar, Anand L, Jeswanth Satyanesan, Senthil Kumar P, Chandrabose Ambedkar MJ, Aravind T, Priyanka Senthil

**Affiliations:** 1 Surgical Gastroenterology, Stanley Medical College, Chennai, IND; 2 Community Medicine, Stanley Medical College, Chennai, IND

**Keywords:** bile duct injury, bile duct stricture, cholecystectomy, hepaticojejunostomy, predictive score, preoperative optimization, risk stratification, stanley bdi-rrs score

## Abstract

Background

Bile duct injury (BDI) remains one of the most serious complications of cholecystectomy and frequently necessitates complex biliary reconstruction. Successful reconstruction depends not only on the anatomical severity of injury but also on multiple physiological, clinical, biochemical, and radiological factors. Existing anatomical classification systems describe the level of injury but do not objectively determine readiness for definitive reconstruction or guide its optimal timing. To our knowledge, no preoperative risk stratification score currently exists for this purpose. We developed the Stanley Bile Duct Injury-Repair Risk Stratification Score (Stanley BDI-RRS) to address this unmet clinical need.

Methods

The Stanley BDI-RRS was developed using a retrospective cohort of patients managed between January 2015 and December 2019. Eight preoperative variables associated with favorable reconstructive outcomes were incorporated into the score: interval from injury to repair ≥6 weeks, absence of vascular injury, bilioma/abscess/biliary fistula, and sepsis/cholangitis, bile duct diameter ≥7 mm, direct bilirubin ≤4.5 mg/dL, serum albumin ≥3.0 g/dL, and Bismuth-Strasberg E1-E2 injuries. A prospective observational internal validation cohort (January 2020-December 2024) included 69 consecutive patients undergoing definitive biliary reconstruction. Patients were assessed using the Stanley BDI-RRS at presentation and, when preoperative optimization was undertaken, reassessed immediately before definitive reconstruction.

Results

Sixty-nine patients were included (mean age 40.8 years); 59 (85.5%) were female, and 10 (14.5%) were male. At presentation, 49 (71.0%) patients had Stanley BDI-RRS scores ≥6, while 20 (29.0%) had scores <6. Eleven (15.9%) patients underwent preoperative optimization, with 10 improving to scores ≥6 before reconstruction. Patients with final preoperative score ≥6 had few adverse postoperative and long-term outcomes, with no postoperative bile leaks or recurrent biliary strictures and one intra-abdominal collection requiring intervention. In contrast, patients with scores <6 accounted for all three postoperative bile leaks (3/69, 4.3%) and the only recurrent biliary stricture (1/69, 1.4%). Overall, 62 patients (89.9%) had an uneventful recovery. Median hospital stay was eight days (IQR, 7-11), and the 18-month stricture-free rate was 98.6% (68/69). No patients required reoperation for postoperative complications during the study period.

Conclusion

The Stanley BDI-RRS score is a novel, simple, objective, and clinically applicable tool for preoperative risk stratification before definitive biliary reconstruction following post-cholecystectomy bile duct injury. By integrating eight readily available preoperative variables, it provides an objective assessment of physiological readiness for definitive biliary reconstruction. This study represents the initial prospective pilot validation of the Stanley BDI-RRS score. Further multicenter external validation with longer follow-up is required before widespread clinical adoption.

## Introduction

Bile duct injury (BDI) remains one of the most serious complications of cholecystectomy [1-3], despite advances in laparoscopic techniques and increasing surgical expertise [2,3]. Although its reported incidence is relatively low (0.3-0.7%) [3], the associated morbidity is substantial, often leading to biliary sepsis, recurrent cholangitis, secondary biliary cirrhosis, repeated interventions, prolonged hospitalization, diminished quality of life, and increased healthcare costs [4,5]. Long-term outcomes depend largely on prompt recognition, appropriate initial management, timely referral to specialized hepatobiliary centers [6,7], and definitive reconstruction performed under optimal conditions. While Roux-en-Y hepaticojejunostomy remains the standard reconstructive procedure [7,8] for major BDI, its success is influenced by multiple patient- and disease-related factors, including the timing of repair, level of injury, bile duct diameter, associated vascular injury, biliary sepsis, liver function, and nutritional status [6-8]. Existing classification systems for postoperative biliary strictures and bile duct injuries, including the Bismuth-Strasberg classification [1,9-11], provide valuable information regarding the anatomical level and extent of injury but do not assess physiological readiness for reconstruction or guide the optimal timing of surgery. Consequently, decisions regarding definitive repair continue to rely largely on individual surgical judgment [12,13] and institutional experience. To address this unmet clinical need, we developed the Stanley Bile Duct Injury-Repair Risk Stratification Score (Stanley BDI-RRS), an objective scoring system that integrates readily available anatomical, clinical, biochemical, and radiological variables associated with successful biliary reconstruction. The present study aimed to validate the Stanley BDI-RRS and assess its role in determining the timing of definitive biliary reconstruction and predicting postoperative and long-term outcomes in patients with post-cholecystectomy bile duct injury.

## Materials and methods

Study design

The Stanley BDI-RRS was developed using a retrospective cohort of patients with post-cholecystectomy bile duct injury managed at our institution between January 2015 and December 2019. Following development of the score, a prospective observational cohort for internal validation was established from January 2020 to December 2024 to validate the score. The study was approved by the Institutional Ethics Committee, Government Stanley Medical College & Hospital, Chennai (Approval No. 2042218).

Statistical development in the derivation cohort

Statistical analysis was performed using IBM SPSS Statistics version 22.0 (IBM Corp., Armonk, NY, USA). The Stanley BDI-RRS was developed using a retrospective cohort of 80 patients with post-cholecystectomy bile duct injury managed at our institution between January 2015 and December 2019. Twenty-three candidate preoperative clinical, biochemical, radiological, anatomical, and surgical variables were evaluated based on the literature review and expert clinical assessment. These included demographic and clinical characteristics, comorbidities, the indication for the initial laparoscopic cholecystectomy, biochemical parameters including serum alkaline phosphatase, aspartate aminotransferase, and alanine aminotransferase, and perioperative and reconstructive variables such as intraoperative blood loss and use of transanastomotic stents. The candidate variables were initially assessed using univariate analysis for their association with favorable reconstructive outcome. Eight variables were selected for incorporation into the Stanley BDI-RRS: interval from injury to repair, associated vascular injury, bilioma/abscess/biliary fistula, sepsis/cholangitis, bile duct diameter, serum albumin, direct bilirubin, and Bismuth-Strasberg level of injury.

Diagnostic performance of variables included in the Stanley BDI-RRS

The diagnostic performance of the selected variables was evaluated in the retrospective derivation cohort (Tables 1-2). Binary and categorical variables, including interval from injury to repair, associated vascular injury, bilioma/abscess/biliary fistula, sepsis/cholangitis, and Bismuth-Strasberg injury level, were evaluated according to their predefined clinical categories. Continuous variables, namely serum albumin, direct bilirubin, and bile duct diameter, were evaluated using receiver operating characteristic (ROC) curve analysis to determine the cutoff values used in the final score. The derived cutoffs were serum albumin ≥3.0 g/dL, direct bilirubin ≤4.5 mg/dL, and bile duct diameter ≥7 mm. The sensitivity, specificity, positive predictive value, negative predictive value, and statistical significance of all eight predictor variables are presented in Table 1.

**Table 1:** Diagnostic performance of candidate predictor variables for favorable reconstructive outcome in the derivation cohort. * Pearson Chi-square; ** Fisher's exact test. PPV, positive predictive value; NPV, negative predictive value.

Parameters	Category	n (%)	Good outcome, n (%)	Poor outcome, n (%)	Sensitivity (%)	Specificity (%)	PPV (%)	NPV (%)	p-value
Interval from injury to repair	≥6 weeks	75 (93.8)	61 (81.3)	14 (18.7)	100	26.3	81.3	100	<0.001**
	<6 weeks	5 (6.3)	0 (0.0)	5 (100.0)					
Associated vascular injury	Absent	75(93.8)	60 (80.0)	15 (20.0)	98.4	21.1	80.0	80.0	0.010**
	Present	5 (6.3)	1 (20.0)	4 (80.0)					
Bilioma/abscess/biliary fistula	Absent	61 (76.3)	51 (83.6)	10 (16.4)	83.6	47.4	83.6	47.4	0.011**
	Present	19 (23.8)	10 (52.6)	9 (47.4)					
Sepsis/cholangitis	Absent	63 (78.8)	56 (88.9)	7 (11.1)	91.8	63.2	88.9	70.6	<0.001**
	Present	17 (21.3)	5 (29.4)	12 (70.6)					
Bile duct diameter	≥7 mm	50 (62.5)	42 (84.0)	8 (16.0)	68.9	57.9	84	36.7	0.035*
	<7 mm	30 (37.5)	19 (63.3)	11 (36.7)					
Serum albumin	≥3.0 g/dL	60 (75.0)	54 (90.0)	6 (10.0)	88.5	68.4	90	65	<0.001**
	<3.0 g/dL	20 (25.0)	7 (35.0)	13 (65.0)					
Direct bilirubin	≤4.5 mg/dL	65 (81.3)	55 (84.6)	10 (15.4)	90.2	47.4	84.6	60	0.001**
	>4.5 mg/dL	15 (18.8)	6 (40.0)	9 (60.0)					
Bismuth-Strasberg classification	E1-E2	35 (43.8)	31 (88.6)	4 (11.4)	50.8	78.9	88.6	33.3	0.022*
	E3-E4	45 (56.3)	30 (66.7)	15 (33.3)					

**Table 2:** Receiver operating characteristic (ROC) analysis of continuous predictor variables. Cutoff values represent the thresholds selected for incorporation into the Stanley Bile Duct Injury-Repair Risk Stratification score. AUC, area under the curve; CI, confidence interval.

Variable	Cutoff	AUC (95% CI)
Serum albumin	≥3.0 g/dL	0.877 (0.800-0.953)
Bile duct diameter	≥7 mm	0.677 (0.532-0.822)
Direct bilirubin	≤4.5 mg/dL	0.729 (0.597-0.860)

**Figure 1:**
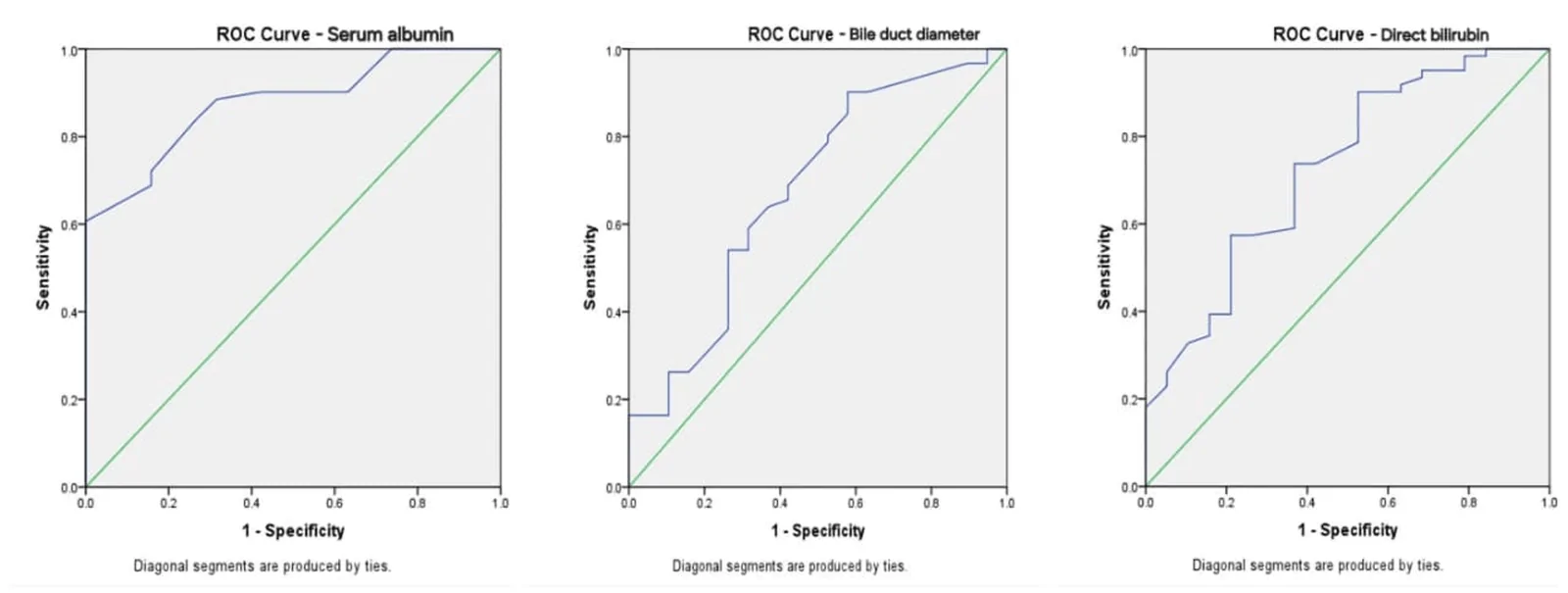
Receiver operating characteristic (ROC) curves for serum albumin, bile duct diameter, and direct bilirubin in the retrospective derivation cohort.

For the three continuous variables, the corresponding ROC curves and area under the curve (AUC) with 95% confidence intervals are presented in Table 2 and Figure 1.

Eight clinically relevant preoperative variables were evaluated using multivariable Firth-penalized logistic regression (Table 3) in the retrospective derivation cohort to assess their independent association with favorable reconstructive outcome. Firth penalization was used to reduce small-sample bias and address sparse-data and separation issues encountered in the analysis. Given the limited number of outcome events relative to the number of candidate predictors, the derivation model may also be susceptible to overfitting, and the resulting estimates should therefore be interpreted cautiously.

**Table 3:** Multivariable Firth-penalized logistic regression analysis of predictors of favorable reconstructive outcome in the derivation cohort.

Predictor	Favorable/good outcome	Unfavorable/bad outcome	Adjusted OR	95% CI	p-value
Interval from injury to repair	≥6 weeks	<6 weeks	13,255.99	31.15-5,641,743.65	0.002
Associated vascular injury	Absent	Present	2.24	0.023-218.47	0.73
Bile duct diameter	≥7 mm	<7 mm	11.81	0.883-157.95	0.062
Direct bilirubin	≤4.5 mg/dL	>4.5 mg/dL	12.17	0.965-153.52	0.053
Serum albumin	≥3.0 g/dL	<3.0 g/dL	17.16	1.396-210.97	0.026
Sepsis/cholangitis	Absent	Present	8.45	0.674-105.77	0.098
Bilioma/abscess/biliary fistula	Absent	Present	23.5	1.591-347.22	0.022
Bismuth-Strasberg classification	E1-E2	E3-E4	8.56	0.586-124.94	0.117

Development of the Stanley Bile Duct Injury-Repair Risk Stratification score

The Stanley BDI-RRS was constructed from the eight variables selected during the retrospective derivation phase (Table 4).

**Table 4:** Components and scoring criteria of the Stanley Bile Duct Injury-Repair Risk Stratification score.

Variable	Favorable criterion	Score
Interval from injury to repair	≥6 weeks from injury	1
Associated vascular injury	Absence of vascular injury	1
Bilioma/abscess/biliary fistula	Absence of bilioma, abscess, or biliary fistula	1
Sepsis/cholangitis	Absence of sepsis or cholangitis	1
Bile duct diameter	≥7 mm	1
Serum albumin	≥3.0 g/dL	1
Direct bilirubin	≤4.5 mg/dL	1
Level of injury	Bismuth-Strasberg E1-E2	1

**Figure 2:**
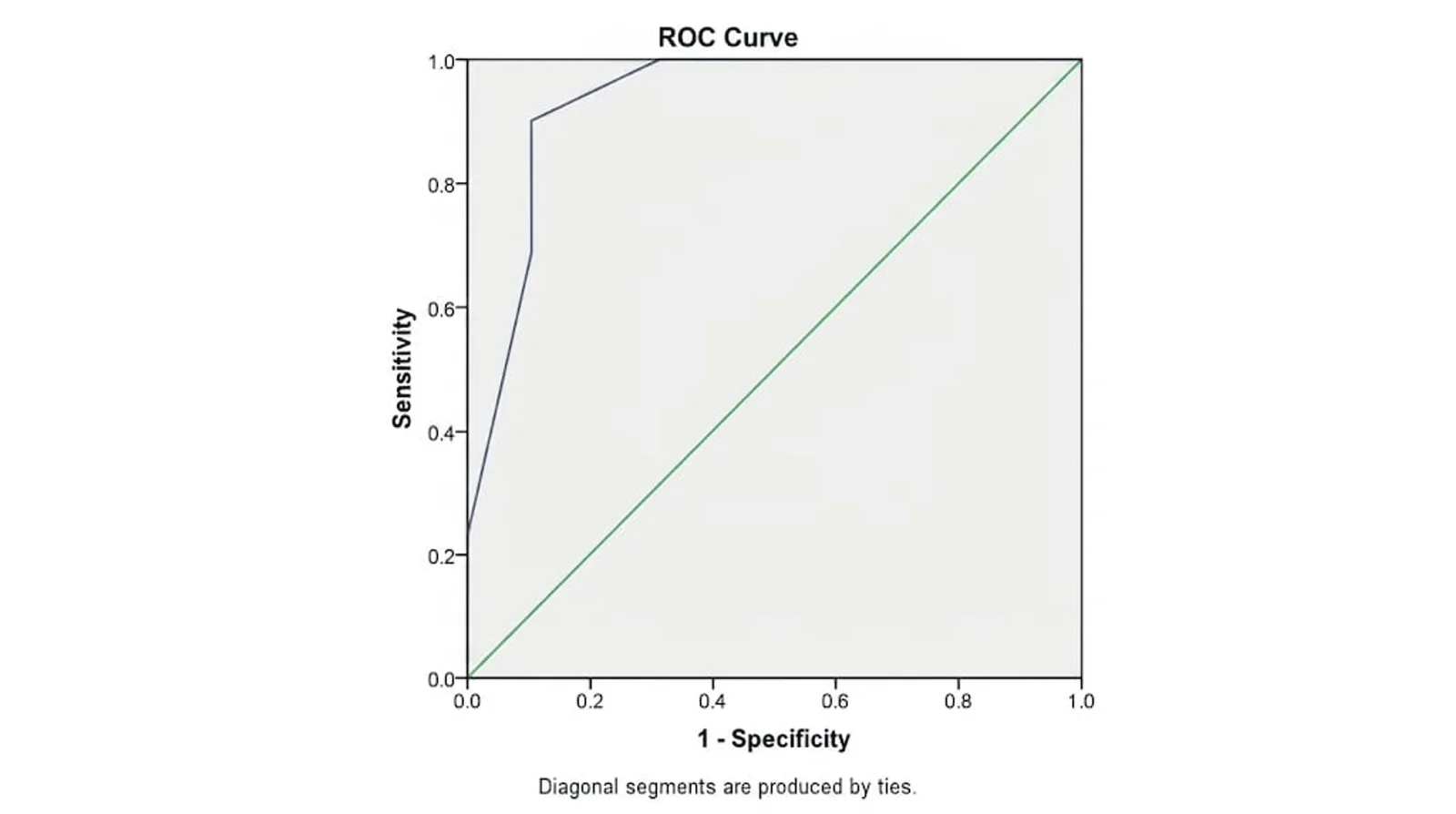
Receiver operating characteristic (ROC) curve demonstrating the performance of the Stanley Bile Duct Injury-Repair Risk Stratification score in the retrospective derivation cohort.

The cutoff values derived from the continuous-variable ROC analyses were incorporated into the score, while the predefined favorable categories were used for the binary and categorical variables. Each favorable parameter was assigned one point and the corresponding unfavorable category zero points, resulting in a total Stanley BDI-RRS ranging from 0 to 8. Equal weighting was used as a pragmatic approach to facilitate bedside application and does not imply that all variables have equivalent biological or clinical significance. Higher scores therefore represent a greater number of favorable preoperative conditions for definitive biliary reconstruction. The predictive performance of the total Stanley BDI-RRS score was also evaluated in the retrospective derivation cohort (n=80; 19 patients with poor outcome) using receiver operating characteristic curve analysis (Figure 2). The score demonstrated an area under the curve of 0.933, indicating excellent discriminative ability. At a cutoff of ≥6, the score showed a sensitivity of 90.2%, specificity of 89.5%, positive predictive value of 96.5%, and negative predictive value of 73.9%.

Objectives

Primary Objective

To prospectively validate the Stanley BDI-RRS score in patients with post-cholecystectomy bile duct injury undergoing definitive biliary reconstruction.

Secondary Objectives

To stratify patients according to the Stanley BDI-RRS and assess the association between the score and postoperative and long-term outcomes.

To evaluate the potential utility of preoperative optimization in improving the Stanley BDI-RRS before definitive biliary reconstruction.

Study population

A total of 69 consecutive patients presenting with bile duct injury were included. The study inclusion and exclusion criteria are summarized in Table 5.

**Table 5:** Study inclusion and exclusion criteria.

Inclusion criteria	Exclusion criteria
Patients with post-cholecystectomy bile duct injury undergoing definitive biliary reconstruction.	Patients with bile duct strictures resulting from causes other than cholecystectomy.
Patients with a minimum postoperative follow-up duration of 18 months.	Patients with malignant biliary strictures.
	Patients with incomplete previous clinical records.
	Patients with a postoperative follow-up duration of less than 18 months or those lost to follow-up.

​​​​​​Preoperative evaluation

All patients were referred to our center from other institutions after initial management. All patients underwent standardized preoperative assessment including complete blood count, liver function tests, serum albumin, ultrasonography with Doppler evaluation, and magnetic resonance cholangiopancreatography (MRCP). These investigations were used to assess the level of injury, bile duct diameter, vascular integrity, presence of bilioma or fistula, and overall physiological status. Associated vascular injury was assessed preoperatively using ultrasonography with Doppler evaluation. No separate intraoperative vascular imaging or systematic intraoperative assessment was undertaken specifically to diagnose vascular injury. Patients with ongoing sepsis, cholangitis, bilioma, abscess, biliary fistula, inadequate biliary drainage, or other potentially modifiable adverse factors underwent appropriate preoperative optimization when clinically indicated. Optimization included drainage procedures such as pigtail drainage, endoscopic biliary stenting, percutaneous transhepatic biliary drainage, or combinations of these interventions, according to the individual clinical circumstances.

Operative technique

All patients underwent definitive biliary reconstruction using a standardized operative protocol performed by experienced hepatobiliary surgeons. Reconstruction was performed using either an end-to-side or side-to-side Roux-en-Y hepaticojejunostomy, according to the operative anatomy. In patients with preoperatively identified vascular injury, the anastomosis was fashioned proximally in well-vascularized biliary tissue, with particular attention to preservation of the collateral blood supply to the anastomotic site. No arterial reconstruction was performed. In proximal or complex injuries where the injury site was difficult to approach because of extensive scarring, the left hepatic duct was widely opened longitudinally to provide adequate biliary exposure and facilitate a wide, well-vascularized anastomosis (Hepp-Couinaud approach) [14]. In high injuries involving the biliary confluence, a parachute technique [15] was used to facilitate precise mucosa-to-mucosa approximation. The biliary-enteric anastomosis was performed using 4-0 polydioxanone (PDS) sutures in a continuous or interrupted fashion, ensuring a tension-free, mucosa-to-mucosa anastomosis and meticulous preservation of the biliary blood supply. An abdominal drain was placed adjacent to the anastomosis at the completion of the procedure.

Outcome measures

A favorable outcome was defined as the absence of postoperative bile leak, intra-abdominal collection requiring intervention (e.g., pigtail drainage), and recurrent biliary stricture during follow-up.

## Results

A total of 69 consecutive patients with post-cholecystectomy bile duct injury who met the predefined eligibility criteria were included in the prospective validation cohort. Their baseline demographic characteristics, injury profile, and previous interventions before referral are summarized in Table 6. The mean age was 40.8 years (range, 21-73 years), with 59 (85.5%) female and 10 (14.5%) male patients. According to the Bismuth-Strasberg classification, eight patients (11.6%) had E1 injuries, 24 (34.8%) had E2 injuries, 31 (44.9%) had E3 injuries, and six (8.7%) had E4 injuries. Before referral to our center, 24 patients (34.8%) had undergone no intervention.

**Table 6:** Baseline characteristics and injury profile (n=69). ERCP, endoscopic retrograde cholangiopancreatography; HJ, hepaticojejunostomy; PTBD, percutaneous transhepatic biliary drainage.

Variable	Value
Mean age (years)	40.8
Age range (years)	21-73
Male	10 (14.5%)
Female	59 (85.5%)
Bismuth-Strasberg classification	
E1	8 (11.6%)
E2	24 (34.8%)
E3	31 (44.9%)
E4	6 (8.7%)
Previous interventions before referral	
No intervention	24 (34.8%)
Pigtail drainage alone	15 (21.7%)
Laparotomy & lavage alone	14 (20.3%)
ERCP + stenting alone	3 (4.3%)
Failed ERCP	2 (2.9%)
ERCP/stenting + pigtail	1 (1.4%)
Laparotomy & Lavage + ERCP/stenting	2 (2.9%)
Previous HJ alone	3 (4.3%)
ERCP/stenting + HJ	1 (1.4%)
Pigtail + HJ	1 (1.4%)
HJ + PTBD	3 (4.3%)

Stanley BDI-RRS scores ranged from 2 to 8. Forty-nine patients (71.0%) had scores of 6-8, whereas 20 (29.0%) had scores below 6 (Table 7).

**Table 7:** Distribution of Stanley Bile Duct Injury-Repair Risk Stratification (BDI-RRS) scores among study participants.

Stanley BDI-RRS score	No. of patients
2	2 (2.9%)
3	6 (8.7%)
4	4 (5.8%)
5	8 (11.6%)
6	11 (15.9%)
7	25 (36.2%)
8	13 (18.8%)
Total	69 (100%)

Eleven (15.9%) patients underwent preoperative optimization procedures, including pigtail drainage (4; 36.4%), ERCP with biliary stenting (3; 27.3%), percutaneous transhepatic biliary drainage (PTBD) (3; 27.3%), and combined pigtail drainage with ERCP (1; 9.1%). Preoperative optimization improved the Stanley BDI-RRS in 10 of the 11 patients (90.9%), resulting in successful conversion to the favorable-risk category (score ≥6) before definitive biliary reconstruction (Table 8).

**Table 8:** Changes in Stanley Bile Duct Injury-Repair Risk Stratification scores following preoperative optimization.

Score	Pre-intervention	Post-intervention
2	1	-
3	6	-
4	3	-
5	1	1
6	-	4
7	-	5
8	-	1

Postoperative and long-term outcomes

Sixty-nine patients underwent definitive biliary reconstruction, including 58 at initial assessment and 11 after preoperative optimization. All patients were followed for 18 months. Patients with Stanley BDI-RRS score ≥6 experienced favorable postoperative and long-term outcomes, with only one of 49 patients (2.0%) developing an intra-abdominal collection, and no patients developed postoperative bile leak or recurrent biliary stricture (0%). In contrast, patients with Stanley BDI-RRS scores <6 experienced higher postoperative morbidity, including bile leak, intra-abdominal collection, and the only case of recurrent biliary stricture observed during follow-up (Table 9). Among patients who underwent preoperative optimization, all 10 patients (100%) who achieved scores ≥6 had uneventful postoperative outcomes without bile leak, intra-abdominal collection, or recurrent biliary stricture.

**Table 9:** Surgical outcomes according to Stanley Bile Duct Injury-Repair Risk Stratification category.

Score category	Patients	Bile leak	Collection	Restricture
Definitive biliary reconstruction				
Scores ≥6	49	-	1	-
Scores <6	9	2	2	1
Definitive biliary reconstruction after optimization				
Scores ≥6	10	-	-	-
Scores <6	1	1	-	-

Overall, definitive biliary reconstruction resulted in favorable postoperative and long-term outcomes, with uneventful recovery in 62 patients (89.9%) and an 18-month stricture-free rate of 98.6% during follow-up (Table 10). All three patients (3/69, 4.3%) who developed postoperative bile leaks were successfully managed conservatively with antibiotics and delayed drain removal following resolution of the leak, with no patient requiring reoperation. Similarly, all three patients (3/69, 4.3%) who developed intra-abdominal collections were successfully managed with ultrasound-guided pigtail catheter drainage and antibiotic therapy, without surgical re-exploration.

**Table 10:** Postoperative and long-term outcomes following definitive biliary reconstruction.

Variable	Value
Hospital stay	
≤7 days	18 (26%)
8-10 days	34 (49%)
>10 days	17 (25%)
Median hospital stay	8 days (IQR 7-11)
Postoperative outcomes	
Uneventful recovery	62/69 (89.9%)
Bile leak	3 (4.3%)
Intra-abdominal collection	3 (4.3%)
Long-term outcomes	
Recurrent biliary stricture	1 (1.4%)
Stricture-free rate at 18 months	68/69 (98.6%)

**Figure 3:**
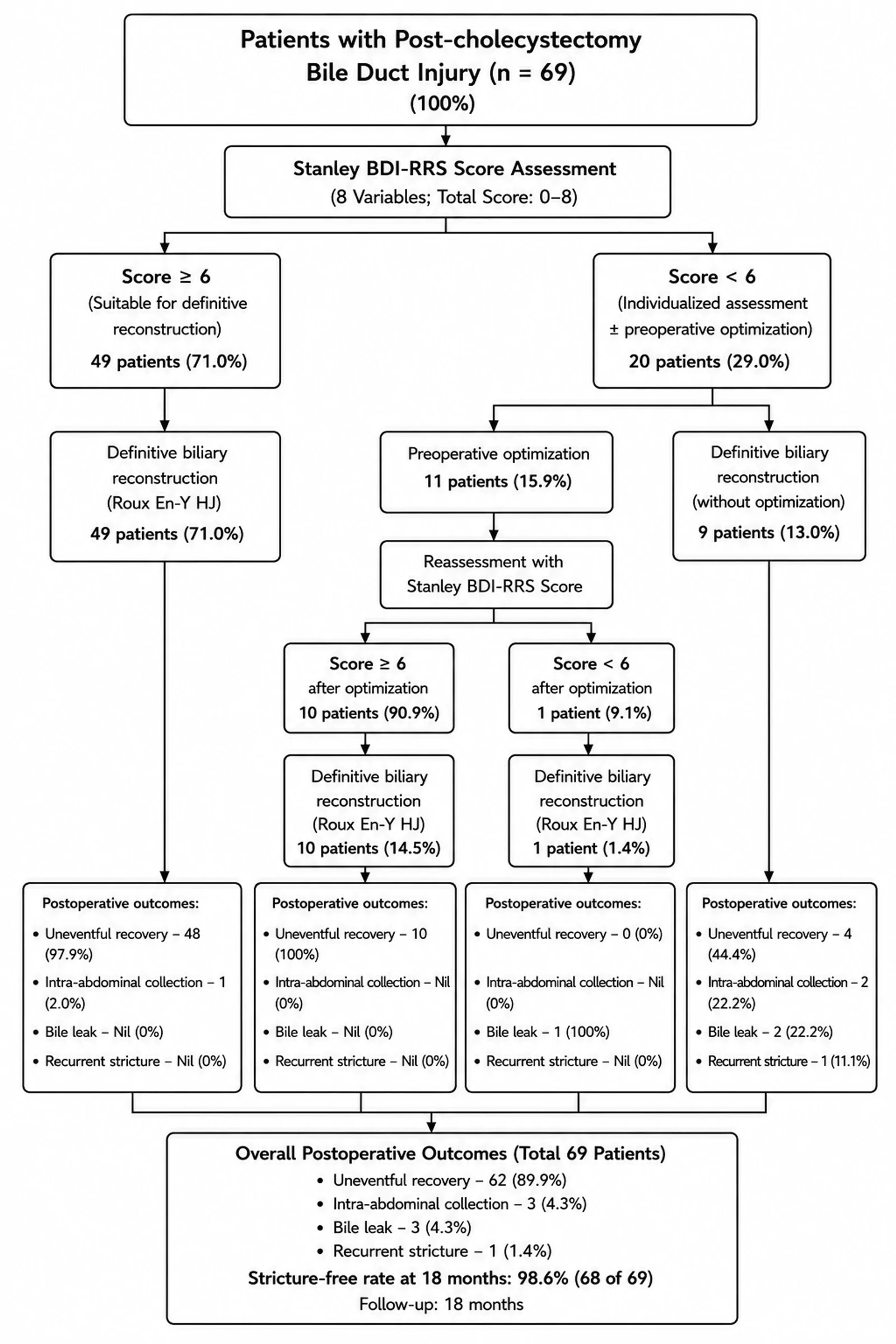
Flow diagram of the prospective internal validation cohort, including preoperative optimization, score reassessment, definitive biliary reconstruction, and outcomes. BDI-RRS, Bile Duct Injury-Repair Risk Stratification.

Figure 3 illustrates the study flow, patient stratification, preoperative optimization, definitive biliary reconstruction, and postoperative outcomes. The median hospital stay was eight days (IQR, 7-11 days), with 18 patients (26%) discharged within seven days, 34 (49%) hospitalized for eight to 10 days, and 17 (25%) requiring hospitalization for more than 10 days. During the 18-month follow-up period, recurrent biliary stricture occurred in one patient (1.4%), resulting in an 18-month stricture-free rate of 98.6% (68/69).

Prospective validation of the Stanley BDI-RRS

**Figure 4:**
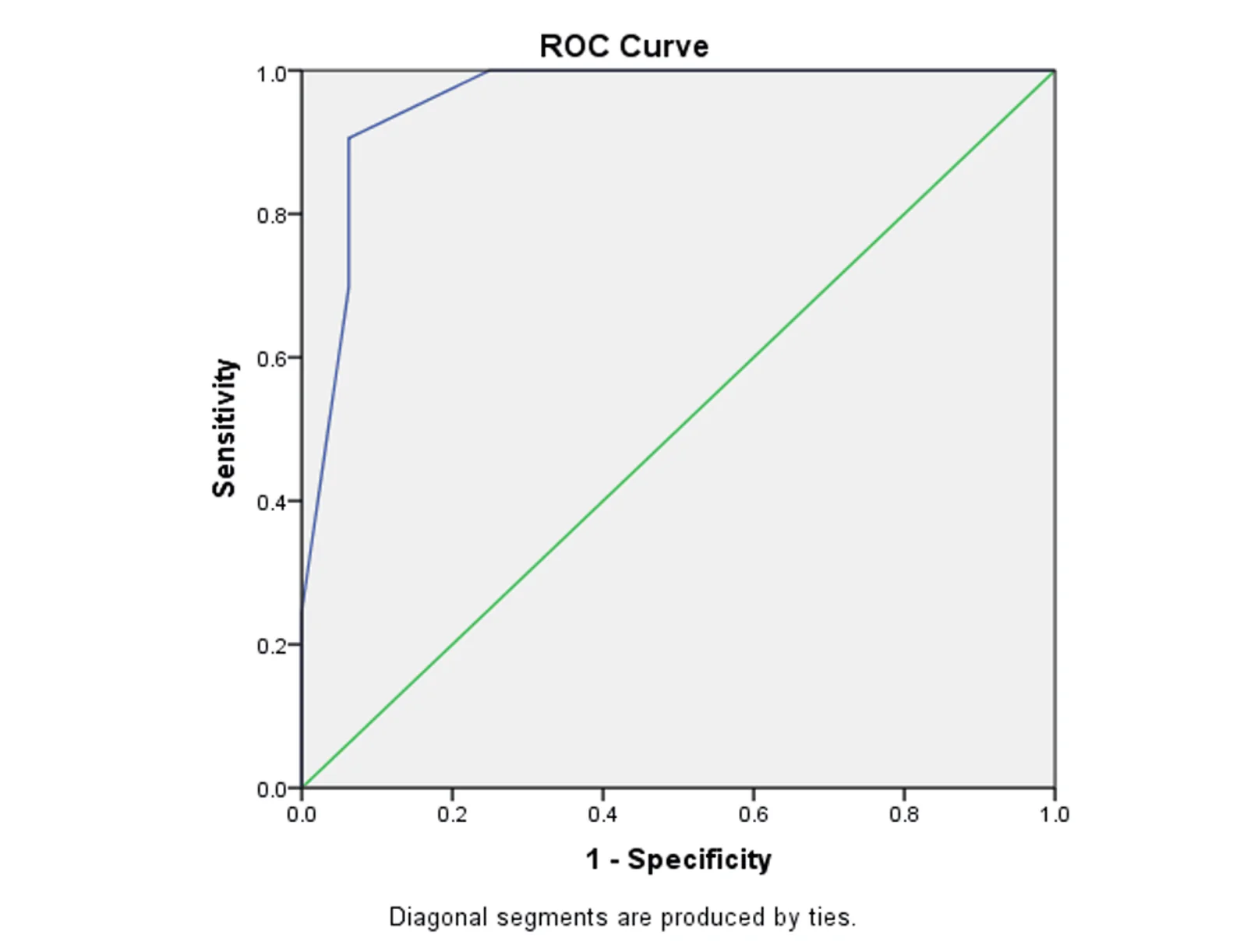
Receiver operating characteristic (ROC) curve demonstrating the performance of the final preoperative Stanley Bile Duct Injury-Repair Risk Stratification score in the prospective internal validation cohort.

All 69 patients underwent definitive biliary reconstruction, including 58 patients at initial assessment and 11 patients following preoperative optimization. For prospective internal validation, the Stanley BDI-RRS was reassessed after preoperative optimization and immediately before definitive biliary reconstruction in patients who underwent optimization. Using the final preoperative score, the Stanley BDI-RRS demonstrated high apparent discriminative performance for favorable reconstructive outcome in the prospective internal validation cohort, with an area under the receiver operating characteristic curve of 0.958 (Figure 4). At the optimal cutoff of ≥6, the sensitivity was 90.6%, specificity was 93.8%, positive predictive value was 98%, and negative predictive value was 75%.

## Discussion

Despite significant advances in laparoscopic cholecystectomy and surgical expertise [1-5], bile duct injury (BDI) remains one of the most devastating complications in hepatobiliary surgery. Although Roux-en-Y hepaticojejunostomy is the gold standard for definitive reconstruction, successful outcomes depend not only on surgical expertise but also on optimal patient preparation and favorable local tissue conditions. Current international guidelines emphasize preoperative optimization [12,13], including control of sepsis, adequate biliary drainage, and referral to experienced hepatobiliary centers before reconstruction. However, no universally accepted preoperative scoring system has been available to objectively assess readiness for definitive repair. The Stanley BDI-RRS was developed to address this unmet clinical need by integrating clinically relevant anatomical, biochemical, radiological, and physiological variables into a simple and objective preoperative risk assessment tool. The bile duct diameter, albumin, and direct bilirubin thresholds used in the score were derived as statistically optimal cutoffs from receiver operating characteristic analysis in the retrospective development cohort, reflecting the best trade-off between sensitivity and specificity for predicting a favorable reconstructive outcome. They should not be interpreted as clinical safety thresholds; patients who meet these favourable score criteria may nevertheless have other infectious, nutritional, or hepatic risks, and standard clinical judgment regarding nutritional and hepatic optimization should continue to apply regardless of score. These findings should therefore be interpreted cautiously and validated in larger cohorts.

The variables incorporated into the Stanley BDI-RRS are supported by evidence from major hepatobiliary centers and international consensus recommendations [12-14]. Delayed referral and repeated interventions may complicate the subsequent management of bile duct injuries and may necessitate complex definitive reconstructive procedures [16]. Delayed reconstruction after resolution of inflammation and sepsis is associated with superior outcomes [6,14,17], while associated vascular injury increases the risk of ischemia and anastomotic failure [18,19]. Persistent bilioma, abscess, and biliary fistula [12] and uncontrolled cholangitis [12,20] indicate ongoing inflammation that adversely affects tissue quality. Serum albumin reflects the patient's nutritional reserve [21]. Preoperative biliary decompression using endoscopic or percutaneous interventions plays an important role in optimizing patients with bile duct injury complicated by cholangitis before definitive biliary reconstruction [22]. Bile duct diameter influences the technical ease and durability of hepaticojejunostomy [23], while lower Bismuth-Strasberg (E1-E2) injuries [1,9-11] are generally associated with less complex reconstruction. Unlike existing classification systems for postoperative biliary strictures and bile duct injuries [1,9-11,24], including the Bismuth-Strasberg classification, which primarily describe the anatomical level and extent of bile duct injury, the Stanley BDI-RRS objectively assesses a patient's readiness for definitive reconstruction by integrating anatomical severity with physiological status, nutritional reserve, biliary decompression, vascular integrity, and timing of repair. By incorporating several clinically modifiable variables, the score may serve as a dynamic tool to support preoperative optimization and assessment of readiness for definitive biliary reconstruction rather than relying solely on a predetermined interval from injury. The individual parameters incorporated into the Stanley BDI-RRS should not be interpreted in isolation, as reconstructive outcome is influenced by their collective contribution. The cumulative Stanley BDI-RRS therefore provides a comprehensive assessment of the overall condition and readiness of the patient for definitive biliary reconstruction.

The present study represents a prospective pilot validation of the Stanley BDI-RRS, a scoring system developed entirely from routinely available preoperative clinical, biochemical, radiological, and anatomical parameters, making it practical and readily applicable in clinical practice. In the prospective internal validation cohort, 49 of 69 patients (71.0%) had Stanley BDI-RRS scores ≥6 at presentation, while 10 of 11 patients (90.9%) who underwent preoperative optimization improved to scores ≥6 before definitive biliary reconstruction. Improvement in the score following preoperative optimization may reflect both the effects of targeted interventions and the passage of time, as an interval of ≥6 weeks from injury to repair is itself a component of the score; therefore, the independent contribution of optimization cannot be determined from the present study. Overall, 62 of 69 patients (89.9%) achieved an uneventful postoperative recovery, and an 18-month stricture-free rate of 98.6% (68/69) was observed during follow-up. Patients with final preoperative Stanley BDI-RRS scores ≥6 demonstrated a low incidence of adverse postoperative and long-term outcomes, with no postoperative bile leaks or recurrent biliary strictures and only one intra-abdominal collection requiring intervention. However, because preoperative optimization was not protocolized and there was no non-optimized control group, the observed improvement in postoperative outcomes cannot be attributed causally to score-guided optimization. Although the findings are encouraging, the score should currently be considered an internally assessed pilot risk-stratification tool rather than definitive evidence of the score's clinical utility.

The principal limitations include the single-center design, relatively small cohort of 69 patients, and limited number of adverse events, which may reduce generalizability and precision of predictive estimates. The small number of adverse outcome events in the prospective internal validation cohort also limits the precision and stability of sensitivity, specificity, predictive values, and long-term outcome estimates, which should therefore be interpreted cautiously. Given the limited number of outcome events relative to the number of candidate predictors, the derivation model may also be susceptible to overfitting. Formal calibration assessment was not performed because of the limited cohort size and sparse outcome events. As a tertiary referral hepatobiliary center with experienced surgeons and a standardized reconstructive protocol, the favorable outcomes observed may partly reflect specialist expertise, referral patterns, and patient selection rather than the score itself. Several score components also overlap with factors routinely considered in clinical assessment of readiness for reconstruction, creating potential incorporation bias; therefore, the score may formalize clinical judgment without yet demonstrating independent clinical utility. Selection bias may also have been introduced by excluding patients with incomplete records, inadequate follow-up, or loss to follow-up. The 18-month follow-up period may also be insufficient to detect late biliary stricture recurrence. The Stanley BDI-RRS has not yet undergone external multicenter validation, and comparison with an established competing model was not possible because no widely accepted preoperative risk-stratification model exists for this setting. Accordingly, the findings should be considered hypothesis-generating. Future studies should externally validate the Stanley BDI-RRS in larger, independent multicenter cohorts and assess its calibration and clinical utility across diverse patient populations. They should also determine whether score-guided optimization improves postoperative and long-term outcomes before widespread clinical implementation.

## Conclusions

The Stanley BDI-RRS score is a novel, simple, and objective preliminary tool that shows promising performance in this prospective internal validation cohort for the preoperative assessment of patients with post-cholecystectomy bile duct injury undergoing definitive biliary reconstruction. By integrating eight readily available anatomical, clinical, biochemical, and radiological variables, the Stanley BDI-RRS score provides a comprehensive assessment of physiological readiness for definitive biliary reconstruction. This study represents the initial prospective internal pilot validation of the Stanley BDI-RRS score. However, external validation in larger multicenter cohorts with longer follow-up is required to establish its reproducibility, predictive performance, and clinical utility before widespread clinical adoption.
